# Comparison of computed radiography and film/screen combination using a contrast‐detail phantom

**DOI:** 10.1120/jacmp.v4i1.2548

**Published:** 2003-01-01

**Authors:** Z. F. Lu, E. L. Nickoloff, J. C. So, A. K. Dutta

**Affiliations:** ^1^ Department of Radiology Columbia University MHB 3‐265, 177 Ft. Washington Avenue New York New York 10032

**Keywords:** computed radiography, contrast‐detail detectability, radiation dose

## Abstract

The purpose of this work is to compare computed radiography (Kodak CR 400) and film/screen combination (Speed 400) systems in regards of patient dose, technique settings, and contrast‐detail detectability. A special contrast‐detail phantom with drilled holes of varying diameter (detail) and varying depth (contrast) was utilized. Various thicknesses of the Lucite sheets were utilized to simulate scattering tissues. Images of the phantom were acquired using a range of 60–120 kVp for film/screen and CR with a conventional x‐ray tube and then for CR with additional 2 mm aluminum added filtration to the x‐ray beam. The patient entrance skin dose was measured while maintaining 1.6 o.d. for film/screen images and 1900 Exposure Index for CR images. CR phantom images were displayed on the diagnostic workstation for soft copy reading as well as printed on films for hard copy reading on viewbox. Four physicists evaluated the images by scoring the threshold target depth along the row of the same target diameter. Detection ratio was calculated by counting the number of detectable targets divided by the total number of targets in the phantom. The overall score was related to the patient entrance skin dose, kVp, and the thickness of the scattering material. The patient entrance skin dose was reduced as the additional aluminum filter was added to the x‐ray beam. Our findings suggested using a higher kVp setting and additional added filtration would reduce the patient entrance skin dose without compromising the contrast‐detail detectability, which was compensated by the contrast manipulation on soft‐copy display workstations. © *2003 American College of Medical Physics.*

PACS number(s): 87.57.–s, 87.62.+n

## I. INTRODUCTION

During the past decade, computed radiography (CR) has gradually gained its widespread acceptance as an alternative method to replace the conventional film/screen combination for digital image acquisition. In comparison to a film/screen combination, a CR system has advantages including a large dynamic range, reduced repeat rates, digital image storage, and image manipulation.^1^–^3^ The disadvantages include reduced spatial resolution and higher start‐up costs.^2^ It has been demonstrated that CR has a linear response over four orders of magnitude of radiation exposure.^1^ This allows CR systems to have a high tolerance for variations in radiation exposures. For certain clinical applications, such as musculoskeletal radiology, in which the image noise can be compromised, the radiation dose for CR images is lower than the dose needed for film/screen.^4^,^5^ However, for the majority of the clinical applications, the radiation dose for CR is greater than the dose for speed 400 film/screen.^3^,^6^,^7^ Therefore, uncertainties remain regarding the optimal exposure techniques for CR with the best possible image quality and the lowest possible patient radiation dose.

Articles have also been published regarding the performance evaluation of a CR system.^7^–^10^

Much of the work has focused on modulation transfer functions, signal‐to‐noise ratio, and detective quantum efficiency analysis, etc. It is shown that the CR systems may have improved low contrast resolution^11^ Threshold contrast detail detectability has been the test to assess such image quality of digital radiographic systems.^12^ The contrast‐detail detectability depends upon various factors such as the noise, radiation exposure level, spatial resolution, contrast resolution, visual response of the observer, etc. Therefore, such tests provide a valuable assessment of the image quality of a digital radiographic system. The ultimate test of the system performance has to involve the diagnostic accuracy using clinical images, which is a difficult and complicated task.

In this article, a contrast‐detail phantom was utilized. A film/screen combination with speed 400 was included for comparison with the CR system. This article studied the relationship of radiation dose, kVp setting, and contrast‐detail detectability of the CR images acquired under various thicknesses of Lucite sheets used to simulate the tissues that generate scattered radiation.

## II. METHODS

**Figure 1:**
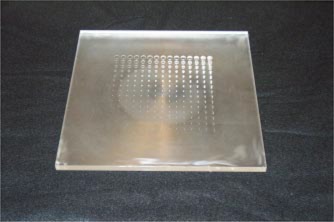
(Color) Picture of the contrast‐detail phantom.

The contrast‐detail phantom^13^ utilized in this study was custom made as shown in Fig. 1. The phantom was constructed on a 1 cm thick Lucite sheet with 26.5 cm×26.5 cm area size. Holes of varying diameters and depths were drilled into the Lucite sheet, 15×15 holes, for a total of 225. The holes in one direction had a constant diameter but decreasing depth (therefore, decreasing contrast on the image). The holes in the other direction had a constant depth (therefore, same contrast on the image) but decreasing diameter. The target diameter ranged from 0.3 to 8 mm and the target depth ranged from 0.3 to 8 mm.

**Figure 2:**
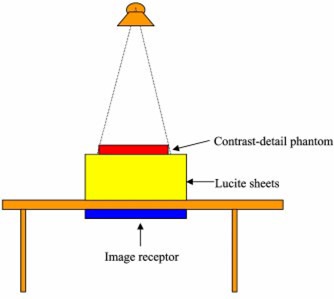
(Color) Diagram of the experimental setup.

We imaged the phantom at the table Bucky as shown in Fig. 2. The grid ratio was 10:1. Additional Lucite sheets were placed underneath the phantom to simulate the tissues that generated scattered radiation. The thickness of these Lucite sheets varied from 5, 10, 15, 20, to 25 cm, simulating different degrees of scattered radiation relative to primary radiation. The x‐ray source to image receptor distance (SID) was initially set at 100 cm for 5 cm Lucite sheets placed underneath the contrast‐detail phantom. The thickness of the Lucite sheets placed underneath the phantom was then increased to generate more scattered radiation. The x‐ray source to image receptor distance (SID) was adjusted in such a way that the geometrical magnification factor for imaging the phantom remained the same no matter what the distance between the phantom and the imaging plate is. The kVp settings varied from 60 to 120 kVp.

The CR system utilized for this study consisted of Kodak GP‐25 PSP plates and a Kodak Model 400 reader. Although this particular Kodak CR model was recently superseded by newer models (CR800 and CR900), the image quality specifications were equivalent for all three systems by design.^14^ The same algorithms were utilized for exposure index, and the same definition was for speed. In addition, the image processing was the same for all three models.^14^ The CR cassette size utilized in this study was 35×43 cm with a matrix size of 2048×2500; thus, the pixel size was 0.17 mm. Before we made the exposure to the CR plates, the entrance exposure to the CR plate after penetrating through the grid was measured. A lead sheet was placed behind the ion chamber to eliminate back‐scattered radiation. By adjusting the mAs, three levels of radiation exposure were determined: 0.5, 1.0, and 1.5 mR. The images were then printed with an Imation Dryview™ laser camera unto film using a fixed window width of 900 and the proper window level adjusted to maintain an optical density of 1.6. The hard copy images were read blindly by four experienced physicists. The threshold depth of the target was scored along each row of the same target diameter. Images were also taken using a speed 400 film/screen system (Fuji HG‐1 intensifying screen and Kodak TMG‐RA‐1 film) for comparison with the images obtained from the CR system. The films were processed in a Kodak M6B processor with seasoned Picker S‐Type chemistry at 35°C. The film density was controlled to be around 1.6 o.d. for all the images on films. The entrance skin exposure (ESE) was measured for all images at the surface of the phantom using a MDH 1015C meter with a 10×5−6 ionization chamber. The ESE was measured free‐in‐air and the back‐scattered radiation from the phantom was not excluded.

In order to study the advantages of using the CR system with higher kVp and more penetrating x‐ray beam, the above tests were repeated on the CR system using extra 2 mm aluminum added filter to harden the x‐ray beam. To take advantage of the data manipulation in image display, the soft copies were read by the four observers on a set of Gray‐scale monitors with a matrix size of 1 k×1.5 k and the maximum luminance of about 180 cd/m^2^. Available tools of the diagnostic workstation were employed to manipulate the digital image data and optimize the image display. The images were scored for the number of visible low contrast objects. The scores obtained from the CR images were compared with the scores obtained from the film/screen images. The entrance skin exposure (ESE) was also measured and compared.

## III. RESULTS AND DISCUSSION

**Figure 3:**
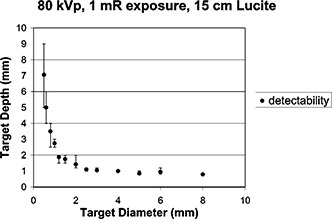
Contrast‐detail detectability curve with 1 mR entrance exposure and 15 cm Lucite as the scattering material and 80 kVp setting. The error bars indicate the varying range of the assessment from four observers.

According to the Rose model,^15^ there is an inverse relationship between the target size and the square of contrast for comparable detection,

$$SNR^{2}=C^{2}NA.$$

where *SNR* is the target signal‐to‐noise ratio, *C* is the target contrast, *N* is the number of detected photons per unit area, and *A* is the target area projected on image. This is true if the image system is ideal. However, in reality, the detectability of a contrast‐detail target is more complicated. We measured the curves of threshold depth versus target diameter under various conditions. As the target size increased, the shallower threshold depth could be visualized; subtler contrast was detectable when the target size was bigger. Figure 3 shows a curve of threshold depth versus target diameter at 80 kVp using a CR system with 1 mR exposure to the CR plate and 15 cm Lucite in the beam to simulate the tissues for scattered radiation. The error bars in this figure indicated the variability range from maximum to minimum scores from four observers.

**Figure 4:**
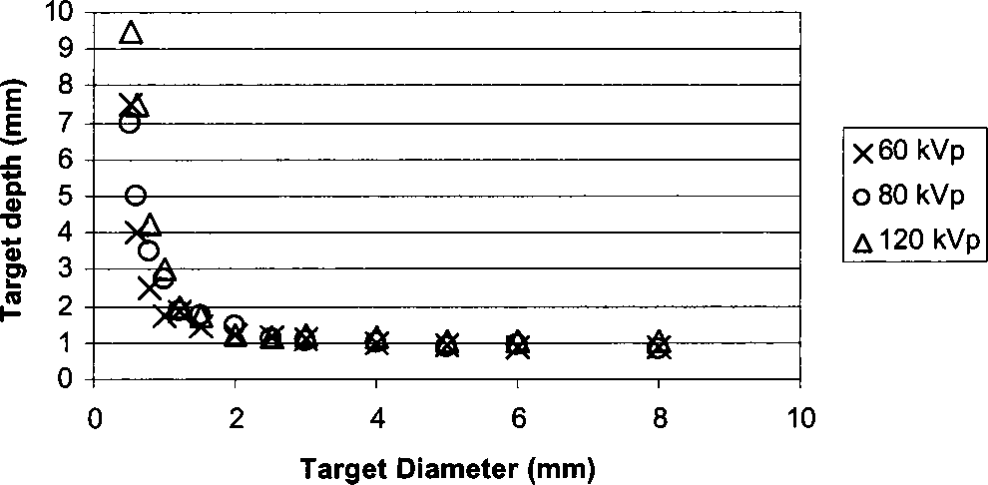
Contrast‐detail detectability curve obtained with a fixed 1 mR entrance exposure to the CR plates and 15 cm Lucite as the scattering material but varying kVp settings from 60–120 kVp.

**Figure 5:**
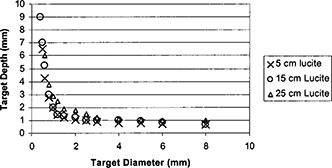
Contrast‐detail detectability curve obtained with a fixed 1 mR entrance exposure to the CR plates and 70 kVp but varying thickness of the scattering material from 5 cm Lucite to 25 cm Lucite sheets.

Figure 4 shows the effect of kVp settings on the contrast‐detail detectability. The curve moving towards the lower left corner indicated better contrast‐detail detectability. As the kVp was reduced, the overall detectability improved. This might be due to the fact that the scatter‐to‐primary radiation ratio reduced with a decreasing kVp. As the scatter‐to‐primary ratio diminished, the image contrast improved. Although the subject contrast improved as the effective x‐ray energy reduced, this improvement was minor because the energy range studied in this article was in the Compton scattering predominant range for Lucite and air. Figure 5 shows the effect of scattering Lucite to the contrast‐detail detectability. The scatter‐to‐primary ratio reduced with a decreasing thickness of the Lucite sheets. Therefore, the contrast‐detail detectability improved with a decrease in the thickness of Lucite sheets.

**Figure 6:**
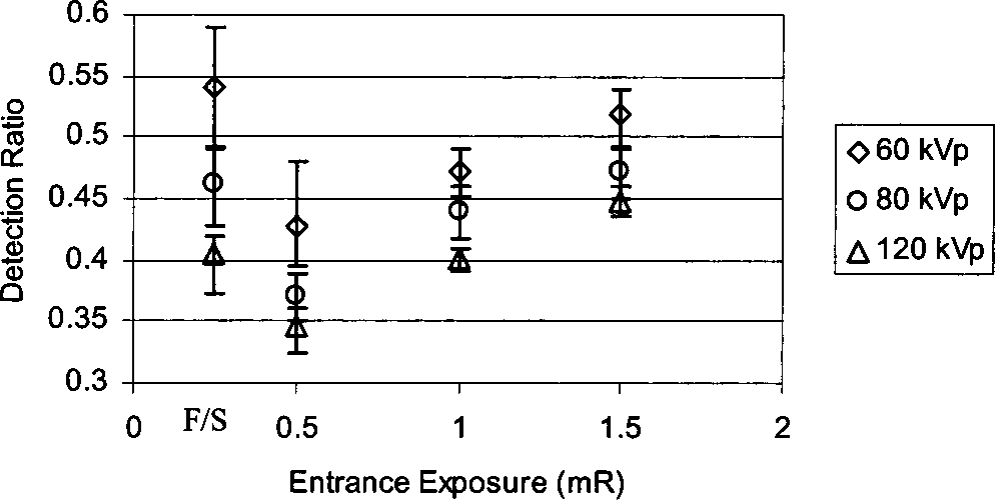
Detection ratio is compared between the film/screen and CR systems. Observer assessments of CR images were performed using hard copies.

To further understand the relationship between the contrast‐detail detectability and the kVp setting, the scattering material thickness, and the radiation to the CR plates, we defined a detection ratio, *R*,

R=n/225,

where *n* was the number of the detected targets assessed by the observer and 225 was the total number of targets on the phantom. Figure 6 shows the average detection ratio obtained from images with 15 cm Lucite sheets underneath the contrast‐detail phantom. The error bars indicate the variability range from maximum to minimum scores from the four observers. The exposure to the CR plates was set at three different values: 0.5, 1.0, and 1.5 mR. Observer assessments of CR images were performed using hard copies for this set of the images. As the exposure to the CR plates increased, the detection ratio increased. Comparing the film/screen system with the CR system, the film/screen had better detection ratio when the exposure to the CR plate was low. However, this margin narrowed as we increased kVp and even reversed when the kVp was very high, as shown in Fig. 6. As the kVp increased, the detection ratio diminished. Nevertheless, this degradation was less significant in CR images than in film/screen images.

For the Kodak CR system, a parameter called the exposure index (EI) was used to indicate the proper signal level of the photo‐simulated phosphor plates. In clinical applications, this exposure index was utilized to monitor whether or not an appropriate radiation level was received by the CR plate. Unfortunately, this exposure index was dependent upon the added filtration of the x‐ray beam. According to the Kodak protocol, the exposure index should be calibrated to 2000 for 1 mR exposure at 80 kVp with a filter of 0.5 mm copper and 1 mm aluminum in the x‐ray beam. The definition of the exposure index was as following under the conditions mentioned above:^16^

Exposure index=1000 log(E)+2000.

**Figure 7:**
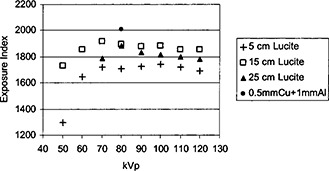
Exposure index for the Kodak CR system is plotted as a function of kVp.

As shown in Fig. 7, the exposure index was measured 2010 on our system at 80 kVp with the Kodak designated filtration and 1 mR exposure. If the filter was changed to 5 cm Lucite sheets, 1 mR exposure to the CR plate produced an image with the exposure index of only 1711. As the thickness of Lucite increased, the exposure index increased. This exposure index was also kVp dependent, as shown in Fig. 7. Therefore, if the exposure index was fixed, the radiation level received by a CR plate varied according to the attenuating materials in the x‐ray beam and kVp settings. For the next experiment, the film density was maintained as close as possible to 1.6 o.d., and the exposure index of the CR images was maintained as close as possible to 1900 for the ESE comparison.

**Figure 8:**
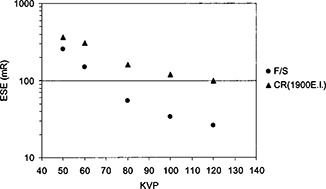
The entrance skin exposure measured at the surface of the phantom is plotted as a function of kVp. The film density was controlled at 1.6 o.d. for the film/screen system and the exposure index was controlled at 1900 units for the CR system. The added filtration was the same for both the film/screen and CR systems.

The entrance skin exposure (ESE) for all the images were measured at the surface of the phantom. Since the nominal relative speed of the CR system^16^ was 200 as compared to the speed 400 film/screen system, the ESE was less for the film/screen system for most images. Figure 8 shows the entrance skin exposure as a function of kVp with 15 cm Lucite.

**Figure 9:**
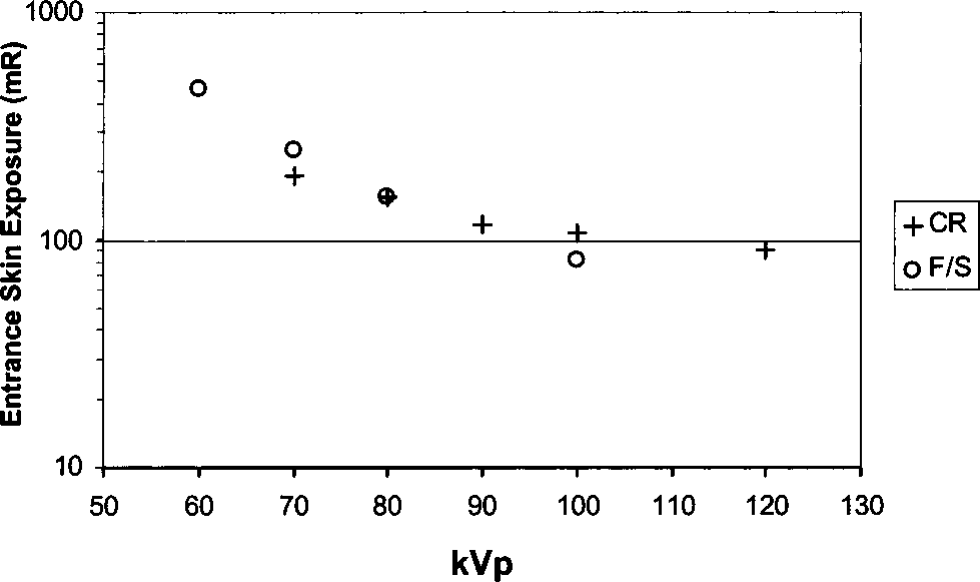
ESE comparison of CR and film/screen systems. An extra 2 mm aluminum filter was added for CR images. Then the ESE for the CR system was similar to the ESE for the film/screen system.

**Figure 10:**
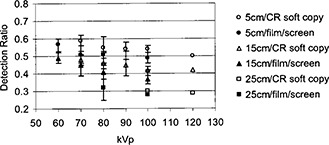
Detection ratio comparison between CR and film/screen systems. Observer assessments of CR images were performed using soft copies, and the tools for image data manipulation and display were used. Different thicknesses of Lucite sheets were used to simulate various degrees of scattered radiation.

In order to reduce the ESE for CR systems, a hardened x‐ray beam was utilized by adding an extra 2 mm aluminum filtration in the beam. As shown in Fig. 9, the ESE for the CR system which had a lower speed was made to be close to the ESE for the film/screen system if an extra 2 mm aluminum filter was added for CR imaging. Therefore, a hardened x‐ray beam was desirable for CR images in order to reduce patient entrance skin exposure. Usually a hardened x‐ray beam compromised the contrast. Nevertheless, soft copy readings of CR images could take advantage of various tools of image data manipulation and image display so that the contrast‐detail detectability would still be acceptable. In Fig. 10, detection ratio *R* was shown against kVp settings from observer assessments on film/screen images and soft copy readings of CR images. Various thicknesses of Lucite sheets were placed underneath the contrast‐detail phantom to simulate different degrees of scattered radiation. The results showed that the detection ratio was better with CR images.

## IV. CONCLUSIONS

The wide dynamic range of a CR system allows a high tolerance for variations in exposure techniques.^1^,^7^ Typically, as we reduce the radiation exposure, the resultant image has more noise. Therefore, optimal exposure techniques are needed to ensure the best image quality at the lowest possible patient exposure. This article compared computed radiography (Kodak CR Model 400) and film/screen combination (speed 400) systems in regards to patient dose, technique settings, and contrast‐detail detectability. We conclude from the above study that (i) the low contrast detectability worsens as the scatter‐to‐primary (*S/P*) radiation increases. *S/P* is directly related to the kVp and the thickness of the Lucite sheets. As the kVp increases, *S/P* increases; thus, the contrast detail detectability reduces. As the thickness of the Lucite sheets increases, *S/P* increases; thus the contrast detail detectability reduces. However, comparing the CR with the film/screen, we find this reduction is less severe in CR images than in film/screen images. (ii) Regarding the radiation dose, the speed of CR plates is approximately 200, which is half of the speed of a regular film/screen combination used in clinical applications, although this speed is highly task dependent. If an extra 2 mm filtration is added for CR imaging, the patient entrance skin dose can be reduced considerably without compromising the contrast‐detail detectability which was compensated by the contrast manipulation on soft‐copy display workstations.

## References

[1] M. Sonoda , M. Takano , J. Miyahara , and H. Kato , “Computed radiography utilizing scanning laser stimulated luminescence,” Radiology 148, 833–838 (1983). 6878707 10.1148/radiology.148.3.6878707

[2] S. R. Kottamasu , L. R. Kuhns , and D. A. Stringer , “Pediatric musculoskeletal computed radiography,” Pediatr. Radiol. 27, 563–575 (1997). 9211947 10.1007/s002470050184

[3] J. A. Seibert , D. K. Shelton , and E. H. Moore , “Computed radiography X‐ray exposure trends,” Acad. Radiol. 3, 313–318 (1996). 8796680 10.1016/s1076-6332(96)80247-9

[4] M. D. Murphey , J. L. Quale , N. L. Martin , J. M. Bramble , L. T. Cook , and S. J. Dwyer III , “Computed radiography in musculoskeletal imaging: state of the art,” Am. J. Roentgenol. 158, 19–27 (1992). 1727344 10.2214/ajr.158.1.1727344

[5] J. Sanfridsson , G. Holje , G. Svahn , L. Ryd , and K. Jonsson , “Radiation dose and image information in computed radiography—a phantom study of angle measurements in the weight‐bearing knee,” Acta Radiol. 41, 310–316 (2000). 10937748 10.1080/028418500127345541

[6] G. C. Weatherburn , S. Bryan , and J. G. Davies , “Comparison of doses for bedside examinations of the chest with conventional screen‐film and computed radiography: results of a randomized controlled trial,” Radiology 217, 707–712 (2000). 11110932 10.1148/radiology.217.3.r00dc12707

[7] W. Huda , L. N. Rill , and A. P. Bruner , “Relative speeds of Kodak computed radiography phosphors and screen‐film systems,” Med. Phys. 24, 1621–1628 (1997). 9350716 10.1118/1.597969

[8] X. Wang , R. Van Metter , D. H. Foos , D. Steklenski , E. M. Perez , E. L. Nickoloff , Z. F. Lu , B. R. Freed , and L. H. Rothenberg , “An automatic quantitative tool for CR image quality testing,” poster presentation on 82nd Annual Meeting of RSNA, 2000.

[9] E. Samei , J. A. Seibert , C. E. Willis , M. J. Flynn , E. Mah , and K. L. Junck , “Performance evaluation of computed radiography systems,” Med. Phys. 28, 361–371 (2001). 11318318 10.1118/1.1350586

[10] C. D. Bradford , W. W. Peppler , and J. T. Dobbins 3rd , “Performance Characteristics of a Kodak Computed Radiography System,” Med. Phys. 26, 27–37 (1999). 9949395 10.1118/1.598781

[11] G. C. Weatherburn and J. G. Davies , “Comparison of film, hard copy computed radiography (CR) and soft copy picture archiving and communication (PACS) systems using a contrast detect test object,” Br. J. Radiol. 72, 853–63 (1999). 10.1259/bjr.72.861.10645191 10645191

[12] A. R. Cowen , A. Workman , and J. S. Price , “Physical aspects of photostimulable phosphor computed radiography [Review],” Br. J. Radiol. 66, 332–345 (1993). 8495288 10.1259/0007-1285-66-784-332

[13] T. Terilli , M. Barnes , A. Dutta , Z. Lu , and E. Nickoloff , “Performance Evaluation of a High‐Strip‐Density Grid Using a Contrast‐Detail Phantom,” Proc. SPIE 3036, 238–252 (1997).

[14] R. L. VanMetter (private communication).

[15] B. H. Hasegawa , The Physics of Medical X‐ray Imaging, 2nd ed. (Medical Physics Publishing, Madison, WI, 1991), Chap. 9.

[16] T. M. Bugucki , D. P. Truernicht , and T. E. Kocher , “Characteristic of a storage phosphor system for medical imaging,” Kodak Publications N‐331 (1995).

